# Supplementary material for: Are carbon emissions trading and green financial instruments synergistic? -Comprehensive quantitative research based on content analysis
Source: PLoS One. 2024 Mar 7;19(3):e0298601. doi: 10.1371/journal.pone.0298601 (PMC10919601; doi:10.1371/journal.pone.0298601)
Supplement: S1 Data — (ZIP) [file pone.0298601.s001.zip › Data/地级市Z_F工作报告.zip!/╔╜╢1⁄2/╚╒╒╒╩╨/└┤╫╘╣1⁄2╓┌║┼í╛┬φ┐╦╩2╛▌═°í┐_2003.docx]

各位代表：

现在，我代表市人民政府向大会作工作报告，请予审议，并请市政协委员和其他列席会议的同志提出意见。

一、过去五年政府工作的回顾

本届政府任期的五年，经历了亚洲金融危机和我国加入WTO等重大事件。面对各种机遇和挑战，市政府紧紧依靠省委、省政府和市委的正确领导，依靠市人大、市政协的监督支持，团结带领全市人民,坚持以邓小平理论和“三个代表”重要思想为指导，解放思想，与时俱进，真抓实干，较好地完成了市十四届人大一次会议确定的任务目标。

经济实力明显增强，结构得到优化。初步测算，2002年实现国内生产总值261.43亿元，比上年增长12.6%，五年平均增长11.9%。农业生产水平不断提高，完成农业增加值53.38亿元，比上年增长3.6%，年均增长4.8%。工业经济快速发展，日照电厂和森博浆纸等一批骨干企业建成投产，全市工业增加值达到91.35亿元，比上年增长16.8%，年均增长15.2%；规模以上工业实现销售收入139.95亿元、利税10.6亿元，年均分别增长17%和18.1%。第三产业繁荣活跃，全市实现第三产业增加值99.8亿元，比上年增长14%，年均增长13.5%；社会消费品零售总额74.91亿元，年均增长12.5%；金融机构存款余额182.51亿元，年均增长15%。地方财政收入达到10.4亿元，按可比口径比上年增长24.04%，年均增长15.3%。三次产业比例调整为20.4：41.1：38.2，二、三产业所占比重分别提高5.5和4.1个百分点；非公有制经济增加值占国内生产总值的比重达到45.5%。

各项改革向纵深推进，对外开放不断扩大。企业产权制度改革不断深化，如期实现了国有大中型企业“三年两个目标，”乡及乡以上企业改制面达到97.2%。坚持用改革的办法解决前进中的问题，“两金”风险得到化解，一些企业的历史遗留问题得到解决。整顿和规范市场经济秩序工作成效明显。机构改革、筛审批制度改革基本完成，农村税费改革取得阶段性成果。广泛开展全民招商活动，努力提高对外开放水平。2002年实际利用外资5583万美元，比上年增长55.1%五年累计实际利用外资5.74亿美元，是前五年的1.8倍；完成进出口总值9.23亿元，比上年增长20.2%，年均增长14.3%，外向度达到29.2%。

基础设施日臻完善，城市面貌发生显著变化。2002年完成固定资产投资85.33亿元，比上年增长13%；五年累计完成固定资产投资350亿元，是前五年的两倍。日照港木片码头、油码头和岚山港液体化工码头竣工投运，港口货物吞吐量和集装箱运量分别达到3805万吨和6.26万标箱。日东高速公路日照段、北沿海路及菏日铁路复线日照段建成通车，同三高速公路、胶新铁路日照段和疏港路开工建设，硬化改造境内国道、省道和县乡公路563公里。沿海防潮堤工程和日照水库除险加固工程进展顺利。城乡电网改造任务全面完成。奎山水厂、市污水处理厂一期工程和莒县污水处理厂投入运行。城市建成区面积扩展到39.9平方公里，新增城市绿地246万平方米，人均公共绿地达到12.4平方米，被评为全国园林绿化先进城市。

科教兴市战略深入实施，社会事业全面发展。五年共取得重要科技成果371项，省级高新技术企业发展到11家。建立了国家农业综合开发高新技术示范园。成立了日照职业技术学院。曲阜师范大学和山东体育学院落户日照，5200余名学生入校学习，结束了我市没有普通高校的历史。新建扩建普通高中17所，高中段招生比例提高22个百分点。市博物馆一期工程、市医院新病房楼建成启用。成功地获得了2005年欧洲级和2006年470级帆船世界锦标赛承办权。计划生育工作连续五年被省委、省政府授予一等奖。国土资源管理得到加强。在全省率先实现了环境保护“一控双达标”，被列为国家可持续发展实验区、全国生态示范区建设试点市和国家环境保护重点城市。新闻出版、民族宗教、外事侨务、对台事务、审计、统计、物价、人防、老龄、史志、档案、气象、残疾人事业等都有新的发展。

城乡居民收入稳步增加，生活质量明显改善。城镇居民人均可支配收入达到7423元，农民人均纯收入3121元，年均分别增长7.4%和6.3%。城乡居民人均住房面积分别达到19和27.1平方米。实现了村村通程控电话，固定电话普及率提高10个百分点，移动电话用户增加22.5万户。社会保障体系不断完善，覆盖面进一步扩大。五年新增城镇从业人员5万人，安置下岗失业职工5923人。

精神文明和民主法制建设不断加强，社会保持稳定。深入开展“三个代表”重要思想学习教育活动，精神文明创建取得明显成效。国防教育和后备力量建设得到加强，双拥共建跨上新台阶，被命名为全国双拥模范城。圆满完成了“三五”普法和第一个依法治市五年规划，推行了村（居）委会直选。反腐败斗争不断深入，一批违法案件得到查处。民主评议行风的经验在全国推广。社会治安综合治理走在全省前列。深入开展“严打”整治和与“法轮功”等邪教组织的斗争，积极做好信访工作，妥善处理人民内部矛盾，强化安全生产监督管理，保持了政治安定和社会稳定。

总之，世纪之交的五年，是我市国发经济持续快速健康发展的五年，是城乡面貌发生显著变化的五年，是人民生活水平不断提高的五年，是社会各项事业全面进步的五年。五年来，我们胜利实现了现代化建设第二步战略目标，初步建立起社会主义市场经济体制，实现了人民生活水平总体上由温饱向小康的跨越。新兴港城日照市正以崭新的姿态，向现代化建设第三步战略目标阔步迈进！

各位代表，过去五年所取得的成就，是党中央、国务院和省委、省政府正确领导的结果，是全市人民同心同德、艰苦奋斗的结果，是在历届市委、市政府打下的良好基础上取得的。在此，我代表市人民政府，向为日照改革开放和现代化建设做出贡献的日照人民，向所有关心、支持和帮助日照发展的同志们、朋友们，表示崇高的敬意和衷心的感谢！

回顾五年来的工作，主要把握了以下几点：一是坚持以解放思想、更新观念为先导。始终把解放思想、更新观念、体制创新作为推动事业发展的根本动力，牢固树立“三个有利于”的标准，促进了经济和社会的发展。二是认真做好结合文章，积极探索符合日照市情的发展路子。确立了港口立市、工业强市、科教兴市、生态建市“四大战略”和招商引资、民营经济、城市建设、旅游经济、园区建设“五项重点”，使加快发展的思路更加清晰，方向更加明确。三是把加快工业化进程作为经济工作的主攻方向，坚持“三改一加强”综合推进，全力加快工业发展，取得了明显成效。四是大力加强精神文明和民主法制建设，深入开展“三讲”教育，不断提高全民思想道德和科学文化素质，为加快发展提供了强大的精神动力和智力支持。五是切实加强政府自身建设，推动政府工作全面 “提速”，妥善解决各种热点难点问题，树立了良好的政府形象。

各位代表，过去五年我们取得了很大成绩，但工作中仍然存在不少困难和问题：经济总量小、结构层次低、竞争能力弱的问题还比较突出；农业承受自然灾害的能力仍很脆弱；工业滞后的问题还没有从根本上得到解决；经济的外向度和非公有制经济比重还比较低；财政增长、农民增收、城市就业的压力仍然很大。对这些困难和问题，我们应高度重视，采取切实有效措施，认真加以解决。

二、本世纪头二十年的奋斗目标和新一届政府的工作任务

根据党的十六大提出的要求，从日照实际出发，本世纪头二十年，我们要全面建设小康社会，努力实现第三步战略目标。到2010年，全市国内生产总值达到700亿元，人均3000美元，主要经济指标基本达到全面建设小康社会的要求；到2020年，国内生产总值达到1960亿元，人均7600美元，达到中等发达国家现在的经济发展水平，基本实现现代化。实现了这个目标，我们的经济社会将更加全面发展、民主法制将更加健全完备、文化科教更加繁荣进步、人民生活将更加幸福美好！

新一届政府任期的五年，是为全面建设小康社会而奋斗的五年，也是为实现第三步战略目标奠定坚实基础的五年。我们要按照“发展要有新思路，改革要有新突破，开放要有新局面，各项工作要有新的举措”的要求，全面做好今后五年的工作，努力实现日照经济的跨越式发展。

经济发展要上新台阶。到2007年，国内生产总值达到482亿元，年均增长13%，人均超过2000美元。地方财政收入达到19亿元，年均增长13%。固定资产投资五年累计完成760亿元，年均增长20%。三次产业比例调整为12：49：39。农业现代化水平显著提高，非农产业劳动力比重达到50%。第二产业增加值达到236亿元，年均增长17%，基本完成向工业化中期阶段的过渡。第三产业增加值达到184亿元，年均增长13%。

改革开放要上新水平。社会主义市场经济体制进一步完善，统一、开放、竞争、有序的市场体系基本形成。非公有制经济比重达到60%以上。进出口总值达到20亿美元，年均增长17%，累计实际利用外资7亿美元，年均增长30%，外向度达到35%。

城市化水平要有新提高。城市建成区面积达到46平方公里、人口达到45万人，城市绿化覆盖率超过45%。全市城镇人口达到130万人，城市化率提高到45%。城市文化繁荣，市民素质有明显提高。

社会事业要有新进步。社会主义民主更加健全，法制更加完备。科技进步对经济增长的贡献率达到55%，人均受教育年限达到10年以上，基本普及高中段教育。办好2005年欧洲级和2006年470级帆船世界锦标赛。人口低生育水平保持稳定，资源综合利用效率明显提高，生态环境更加优美，建成国家卫生城市和国家环保模范城市。

人民生活要有新改善。城镇居民人均可支配收入达到1万元，农民人均纯收入达到4200元。城镇登记失业率控制在4%以内。社会保障体系进一步完善。城乡居民的衣食住行条件进一步改善，人均期望寿命达到74岁。

各位代表！日照作为一座新兴的沿海港口城市，拥有得天独厚的区位优势和环境优势，也拥有巨大的发展潜力和广阔的发展前景。我们坚信，只要认真贯彻落实“三个代表”重要思想，抢抓机遇，开拓创新，真抓实干，我们的奋斗目标就一定能够实现！

三、2003年经济和社会发展的主要任务

今年是深入贯彻十六大精神的第一年，也是新一届政府工作的开局年，做好今年的工作至关重要。政府工作总的要求是：以邓小平理论和“三个代表”的重要思想为指导，以加快发展为主题，突出抓好更新观念、扩大开放、结构调整三个关键环节，全力推进市场化、国际化、城市化“三化”进程，认真实施港口立市、工业强市、科教兴市、生态建市“四大战略”，继续抓好招商引资、民营经济、城市建设、旅游经济、园区建设“五项重点”，推动日照经济的跨越式发展和社会全面进步。

经济和社会发展的主人预期目标是：国内生产总值增长13%，人均超过1万元；三次产业比例调整到18：43：39；地方财政收入增长12%；全社会固定资产投资增长20%；社会消费品零售总额增长13%；进出口总值增长15%；合同利用外资、实际利用外资分别增长50%和40%；城镇居民人均可支配收入和农民人均纯收入分别增长7%和4%；城镇登记失业率控制在3.5%以内；人中自然增长率控制在6%以内。

（一）进一步强化农业的基础地位，提高农业和农村经济发展水平。要把解决“三农”问题摆到更加突出的位置，更多地支持农业、关心农民、关注农村。要以增加农民收入为主题，突出抓好农村经济发展，开创农业和农村工作的新局面。

大力调整农业和农村经济结构。要在保证粮食安全的前提下，按照“调优、调特、调强”的思路，大力调整种植业结构，发展高效农业、特色农业、观光农业和创汇税农业，年内新增优质高效经济作物10万亩。农业内部结构调整，要大力发展海洋经济，抓好畜禽养殖，尽快使水产业、畜牧业的比重超过和赶上种植业。农村经济结构调整，要着重发展农村二、三产业，扩大劳务输出，促进农村经济发展。

努力提高农业产业化水平。对龙头企业加大扶持力度，尽快做大做强，努力培育出一大批能带动产业化发展的龙头企业。积极扶持发展各类合作经济组织，带动农业的专业化生产、区域化种植、产业化经营、规模化发展。要加快建立与国际接轨的农产品质量标准体系，大力发展无公害农产品、绿色食品和有机食品，抓好10万亩放心菜、10万亩放心茶和5万亩放心果基地建设，提高农产品市场竞争力。

加快城镇化建设步伐，促进乡镇经济发展。按照“科学规划、合理布局、体现特色、注重效益”的原则，加快农村小城镇建设。引导乡镇搞好工业园区和专业批发市场建设，提高城乡化水平，促进农村二、三产业向城镇聚集，加快农村劳动力向非农产业转移，走通过减少农民来富裕农民的路子。

加强农业基础设施和服务体系建设。抓好日照、青峰岭水库除险加固和日照水库灌区配套等重点水利工程，新增节水灌溉面积5.4万亩。继续实施小流域治理、封山育林、平原绿化和“三沿一环”绿化工程，改善农村生态环境。抓好建设国债向农村倾斜的机遇，加快乡村道路改造。健全农业社会化服务体系，做好100个村、1000个农户的科技帮扶工作，提高农业科技水平。

继续深化农村改革。认真落实《农村土地承包法》，稳定土地承包关系。引导有条件的地方积极稳妥地推行土地使用权流转，促进规模经营。继续巩固农村税费改革成果，积极发展壮大农村集体经济，切实减轻农民负担。

（二）进一步突出工业的主导地位，全力加快工业发展。要牢固树立“工业强市”的思想，走新型工业化路予，以膨胀规模、优化结构、提高效益为主攻方向，以培育优势产业和骨干企业为抓手，推动工业经济快速发展。

加快培育优势产业。依托日照、岚山两个天然良港，大力培植以治金、粮油加工、液体化工、木制品加工为主的临海工业，实行倾斜扶持政策，着力将其培大培强，力争经过几年的努力，培育出年销售收入过500亿元的行业和过100亿元的企业。年内重点抓好投资20亿元的500万砘H型钢一期工程等13个投资亿元以上的大项目建设，使工业项目建设投资达到60亿元以上。抓好三维油脂、新粮油脂、岚桥集团木制品加工、凌云海糖业、喜多来彩涂钢板等一批投资超亿元的骨干项目投产。加快推进森博浆纸二期、上海钢铁、电解铝等大项目的洽谈和建设进程。要积极运用高新技术和信息手段，改造机械、食品、建材、纺织服装等传统产业，使之焕发新的生机和活力。加快培育高新技术产业，年内重点抓好鼎新玻璃公司300万套投影显示玻壳等13个高新技术项目的建设，高新技术产值占规模以上工业产值的比重提高2个百分点。

集中力量培育优势骨干企业。鼓励重点骨干企业积极与国内外大企业、大集团联合重组，加大技术改造力度，努力做大做强。年内力争使销售收入过10亿元的企业由2家发展到4家、过亿元的由31家发展到41家；利税超过5000万元的由2家发展到6家，过千万元的由25家发展到35家。继续滚动实施百项工业重点项目，突出抓好华龙纺织集团高档喷气织机等90个投资过千万元的技改项目，全年完成技改投入25亿元。

进一步深化企业产权制度改革，加快建立现代化企业制度。加强对已改制企业的监督管理，完善法人治理结构，实现规范动作。抓好市级重点骨干企业集团的公司制改革，指导建立规范的现代企业制度。积极争取国家政策性破产，使劣势企业尽快退出市场。加强企业管理人员、技术人员、营销人员三支队伍建设，提高企业营销管理水平。

民营企业是我市经济发展最具活力的重要力量。要认真落实《中小企业促进法》和各项扶持鼓励政策，为民营经济发展创新良好的舆论环境、政策环境和法律环境，促其快速健康发展。引导民营企业加快技术改造和管理创新，提高发展水平。搞好民营经济园区配套建设，走集约化发展的路子。年内新增民营业户1万户以上，民营经济增加值、利税均增长30%以上，民营经济增加值占国内生产总值的比重达到33%。

（三）大力发展第三产业，培育新的经济增长点。要树立大旅游意识，加快旅游业的发展。以创建中国优秀旅游城市为目标，以景区景点开发建设为重点，坚持“吃、住、行、游、购、娱”配套建设社会力量投资旅游业，加快灯塔广场、海洋生物馆、太公乐园及浮来山、五莲山、阿掖山等景区景点建设，增强吸引力。搞好步行商业街、星级宾馆等旅游设施配套，抓好旅游商品、纪念品和特色餐饮的开发，提高旅游客源市场。加大旅游环境整治力度，规范旅游市场秩序，树立海滨旅游城市的良好形象。

大力发展港口经济，全力做好以港兴市的文章。港口是日照发展的优势所在，活力所在，希望所在，港口兴则日照兴。要认真搞好日照港、岚山港的发展规划，加快两港的建设步伐，全力推进日照港东港区三期、集装箱码头、粮食码头和岚山港通用泊位建设，年内港口货物吞吐量超过4000万吨、集装箱运量达到9万标箱，力争五年内实现港口货物吞吐量1亿吨、集装箱运量50万标箱的目标。完善港路配套的大交通体系，加快同三高速公路和胶新铁路日照段工程建设，确保年内竣工通车。要借助港路交通优势，突出连锁经营、物流配送、批发市场三个重点，加强物流基础设施建设，加快培育现代物流业，尽快建设一处大型物流中心。

积极发展金融业。要调整优化信贷结构，加大对经济发展的信贷支持力度。继续开展创建金融安全区工作，加强金融监管，积极清收不良贷款，保证经济和金融安全运行。进一步培育和规范房地产市场，促进房地产业快速健康发展。加快发展信息咨询、社会中介等新兴服务业，引导居民扩大汽车、旅游、通信、文化、体育等方面的消费，培育新的消费热点。

（四）加快经济国际化进程，全面提高对外开放水平。牢固树立“投资环境是经济发展生命线”的意识，全力改善投资环境。在加强硬环境建设的同时，大力强化软环境建设，在思想观念上、政策法规上和运作方式上自觉与国际市场接轨，建立起能够适应经济国际化要求的发展环境。加强社会信用建设，整顿和规范市场经济秩序，切实保护投资者的合法权益，尊重不同投资者的文化传统和风俗习惯，建立起能够适应外商工作和生活的“仿真”环境。

大力发展加工贸易。要把加工贸易作为发展外向型经济的突破口，紧紧抓住不放，组织动员全社会的力量积极兴办加工贸易项目，努力把我市建成日本、韩国等周边国家和地区的加工基地、装配基地、出口基地，年内加工贸易额增长50%以上。加快保税仓库建设，积极争取建立国家级出口加工区，为发展加工贸易创造良好的条件。进一步调整优化出口商品结构，开拓国际市场，积极开展对外工程承包和劳务输出，保持对外经济贸易的快速增长。

进一步落实招商引资责任制，努力实现招商引资的新突破。全年完成招商引资任务54亿元，比上年增长30%以上。抓住当前国际产业转移加快、国内民间资本投资热潮兴起的有利时机，积极争引跨国公司、上市公司特别是世界500强企业到日照投资。对引进的重大项目继续实行项目推进组的方式，加大跟踪服务力度，促其尽快落地建设。在继续组织好全民招商的同时，抓好以商招商、委托招商和专题招商，提高招商成效。要抓好日照经济开发区等各类园区的建设和发展，使其成为招商引资的载体、对外开放的主体、外商投资的热土。

（五）搞好城市基础设施建设，加快推进城市化进程。

继续抓好城市绿化、美化、亮化，加快“三山五河”治理，新增城市绿化面积120万平方米。高水平地搞好万平口区域开发建设，使之成为山东沿海的新亮点。完成市广播电视中心、火车站及站前广场改造工程，加快垃圾处理厂、污水处理厂二期和城市燃气管网工程建设，进一步完善城市功能。加快旧城改造步伐，逐步解决“城中村”的问题。围绕“一个中心、两个县域中心、八个中心镇”的总体格局，搞好县城和小城镇规划建设，带动县域经济发展。

搞好城市管理和经营。完善城市管理体制，抓好城市环境综合整治，切实解决城市管理滞后的问题，提高城市管理水平。加快城市公用事业经营体制改革，推进市政基础设施经营权转让，拓宽城市建设投融资渠道。深化城市土地使用制度改革，全面推行土地收购储备和招标拍卖制度，保证政府高度垄断土地一级市场，规范二级市场，放开三级市场。推进户籍制度改革，实现城乡劳动力的合理流动。加快城市经济发展，膨胀城市经济规模，充分发挥城市对经济发展的推动作用和辐射作用。

加强城市社区建设。按照便于服务管理、便于资源共享、便于居民自治的原则，建立完善社区服务体系，努力形成一批环境优美、管理规范、设施配套、文化繁荣的新型社区，以社区文明促进城市文明。

（六）努力增加财政收入，不断改善群众生活。把培植财源作为经济工作的重点立足点，大力培植高税产业，不断优化财政收入结构，确保财政收入稳定增长。盘活国有资产，增加变现收入，实现财政资金的良性循环。坚持依法治税，严厉打击涉税违法犯罪行为，确保各项收入及时足额入库。继续扩大“收支两条线”管理范围，规范“票款分离”制度，实行预算内外资金综合使用。进一步调整优化支出结构，大力压缩一般性开支，集中财力保工资、保稳定、保法定支出。按照转变政府职能和建立公共财政的要求，清理财政供给范围，减少财政供养人员。进一步完善政府采购制度，提高财政资金使用效益。

千方百计改善群众生活。认真落实促进就业和再就业的政策措施，努力拓宽就为渠道，年内新增就为3.4万人，组织农村劳动力跨区域就业7200人。强化社会保险基金管理，确保企业离退休人员养老金按时足额发放，确保符合条件的失业人员按时足额领到失业保险金，确保符合条件的城市居民全部享受最低生活保障，农村最低生活保障对象达到1.2万人。建设奎山供水水源二期、岚山调水、两城河向市区调水和五莲县城调水工程，解决20万城市居民生产生活用水问题。加快农村人畜饮水工程建设，彻底解决42个历史性缺水村、1.24万人的饮水困难。

（七）加强政治文明和精神文明的建设，推进民主法制进程。坚持用十六大精神和“三个代表”重要思想武装广大干部群众，引导人们树立中国特色社会主义共同理想。广泛开展精神文明创建活动，提高人们的思想道德水平。要着力培植日照的城市文化，将日照的古文化与现代文化相结合，努力培育具有时代特色的社会价值取向和生活理念，并以此引导和规范人们的思想行为，增强现代市民意识，提高市民的整体素质。加强国防教育和民兵预备役建设，落实优抚安置政策，积极开展军民共建活动，争创全国双拥模范城二连冠。

加强民主政治建设，推进依法治市。积极推行民主选举、民主决策、民主管理、民主监督和政务、厂务、村务公开，依法保障人民群众的民主权利。继续坚持民主评议行风制度。广泛接受社会各界的监督。主动加强与民主党派、工商联、无党派人士和人民团体的联系，征求他们对政府工作的意见和建议。全面实施“四五”普法和第二个依法治市五年规划，提高全民法律素质，特别是增强各级党委领导干部的法制观念和依法办事能力。

努力维护社会稳定。加强社会治安综合治理，深入开展“严打”整治斗争，严厉打击各种犯罪行为，不断增强人民群众的安全感；坚持不懈地开展同“法轮功等邪教组织的斗争；高度重视和正确处理人民内部矛盾，努力把不稳定因素解决在基层、解决在萌芽状态；严格落实安全生产责任制和事故责任追究制，加大专项整治力度，防止各类重大安全责任事故的发生，巩固发展我市安定团结的政治局面与和谐稳定的社会环境。

（八）积极实施科教兴市和可持续发展战略，发展各项社会事业。深化科技体制改革，加快构建以企业为主体，以科技园区为依托，以产学研合作、中介服务、风险投资为支撑的科技创新体系和技术推广服务体系。加快大学科技园建设，年内完成曲阜师范大学日照校区和山东体育学院二期工程，搞好济宁医学院、山东水利职业学院一期工程建设，在校大学生达到2万人。加快清华大学、北京大学等大专院校和科研单位研发基地的建设，促进科技成果转化。搞好科技推广普及，提高全民科学文化素质。积极培养和引进各类人才，用好现有人才，形成尊重知识、尊重人才的良好社会风尚。落实教育优先发展地位，改革完善教育管理体制和中小学学制，调整优化学校布局，全面完成危房改造任务。积极鼓励社会力量办学，加快普及高中段教育，高中段招生人数增长12%。大力发展职业教育和成人教育，为经济建设培养适用人才。

认真落实三项基本国策。全面推进计划生育优质服务，稳定低生育水平，提高出生人口素质。加强国土资源管理，保持耕地动态平衡。推行海域使用许可证和有偿使用制度，依法保护海洋资源。加强水资源统一调配和科学管理，实行计划用水制度，严格控制沿海和城区地下水开采，积极推行中水回用，建设节水型社会。全面落实环境目标责任制，严格执行环境保护“一票否决”和“三同时”制度，积极发展循环经济，推行清洁生产，争创国家可持续发展示范区。

积极发展文化事业和新闻出版事业，繁荣文艺创作，加强文化市场管理，活跃城乡文化生活。完成广播电视体制改革，加快有线电视网络建设，年内有线电视入村率力争达到30%。加强卫生基础设施建设，深化医疗卫生体制改革，提高城乡医疗服务水平。做好世界帆船锦标赛的前期筹备工作。

四、加强政府自身建设

加强政府自身建设，是做好政府工作的重要保证。新一届政府要在市委的领导下，自觉接受人大的法律监督、工作监督和政协的民主监督，按照“三个代表”的要求，在加强自身建设上要有新的突破。

要把发展作为第一要务，努力把新一届政府建设成为干事创业的政府。加快发展是时代的要求、人民的期盼、政府工作的重中之重。新一届政府要始终把发展摆在首位，以振兴日照为已任，聚精会神搞建设，一心一意谋发展。要大力弘扬艰苦创业、真抓实干的精神，旗帜鲜明地支持鼓励创业者、关心爱护改革者、批评教育空谈者、追究惩治诬陷者，努力营造干事创业的良好氛围；大力弘扬敢为人先、争创一流的精神。千方百计加快发展速度，经过五年的努力，使日照经济发展水平在全省地级市中的位次有所上升；大力弘扬与时俱进、开拓创新的精神，坚持用市场经济的手段抓经济工作，用创新的精神培植发展新优势，使日照的经济结构更趋合理，现代化水平明显提高。

牢固树立公仆意识，努力把新一届政府建设成为一心为民的政府。我们的政府是人民的政府，全心全意为人民谋利益，是政府工作的根本出发点和落脚点。新一届政府要时刻把群众的冷暖疾苦挂在心上，深怀爱民之心，恪守为民之责，善谋富民之策，多办利民之事，精心组织实施好市委提出的五项“为民工程”，以实际行动密切同人民群众的血肉联系。要建立健全深入了解民情、充分反映民意、广泛集中民智、切实珍惜民力的决策机制，实行重大决策听证制度、公众参与制度和政务公开制度，使政府工作更加顺民心、合民意。

加快转变行政职能，努力把新一届政府建设成为务实高效的政府。适应发展社会主义市场经济的要求，积极推进政企分开、政事分开，使政府职能真正转到经济调节、市场监管、社会管理和公共服务上来。进一步规范审批行为，推行“一站式”审批和电子政务，年内建成市级行政审批服务中心。要按照“马上就办”的要求，推动政府工作全面“提速”。坚持求真务实的工作作风，不做表面文章，不摆花架子，集中精力抓推进、抓落实，确保抓出效果、干出水平。

坚持严格执法，严格自律，努力把新一届政府建设成为公正廉洁的政府。认真贯彻依法治国方略，严格坚持依法行政，严厉查处徇私枉法行为，依法维护社会公正。大力弘扬艰苦奋斗的优良传统作风，坚决反对讲排场、比阔气，狠刹奢侈浪费之风，勤俭办一切事业。要模范遵守廉洁从政的各项规定，认真履行党风廉政建设责任制。政府各级领导干部不仅要带头廉洁自律，还要带好班子和队伍，管好亲属及身边工作人员。要坚持从严治政，加大惩治腐败的力度，对违法违纪行为要认真追究和惩处，决不姑息，对腐败案件和腐败分子，要一查到底，严惩不贷！

各位代表！

全面建设小康社会，基本实现现代化，是时代赋予我们的庄严使命。让我们紧密团结在以胡锦涛同志为总书记的党中央周围，高举邓小平理论伟大旗帜，全面贯彻十六大精神和“三个代表”重要思想，在市委的正确领导下，同心同德，发奋图强，努力开创日照更加美好的未来！
